# Supplementary material for: Tiny droplets of ocean island basalts unveil Earth’s deep chlorine cycle
Source: Nat Commun. 2019 Jan 4;10:60. doi: 10.1038/s41467-018-07955-8 (PMC6320363; doi:10.1038/s41467-018-07955-8)
Supplement: Supplementary file 1 — Supplementary Information [file 41467_2018_7955_MOESM1_ESM.pdf]

Supplementary Information

Tiny droplets of ocean island basalts unveil Earth's deep chlorine cycle

Hanyu et al.

Supplementary Note 1:

Chlorine enrichment: Assimilation versus source characteristics

The basalts in oceanic settings are susceptible to Cl contamination because they are in contact with seawater. Submarine quenched glasses occasionally show anomalous Cl enrichment[1-8]. One advantage of analysis of olivine-hosted melt inclusions is that they are less likely to have been contaminated by seawater during eruption because they are enclosed in olivine phenocrysts. Nevertheless, Cl contamination of melt inclusions cannot be fully ruled out if magmas in crustal levels assimilate hydrothermal brine and brine-impregnated oceanic crust before melt inclusions are trapped in olivine phenocrysts. Previous research[2] suggested possible Cl contamination based on volatile and lithophile element compositions of olivine-hosted melt inclusions in Raivavae basalts. That study classified inclusions based on Cl concentration and Cl/K into the three following types: Type-1 inclusions have relatively low Cl concentrations (<600 p.p.m.) and low Cl/K (~0.03); type-2 inclusions have higher Cl concentrations (400–1400 p.p.m.) and Cl/K (0.04–0.11) than type-1 inclusions; and type-3 inclusions show extreme Cl enrichment (>1 wt%) despite having K concentration ranges that are similar to those of type-1 and type-2 inclusions. Chlorine enrichment in type-2 and type-3 inclusions can be attributed to brine assimilation and anatexis of brine-impregnated oceanic crust, respectively[2].

We did not find any highly Cl-rich melt inclusions equivalent to type-3 inclusions in our sample sets. Nevertheless, some inclusions in the Rairua and Anatonu basalts might have been affected by assimilation of shallow contaminants that included Cl. In incompatible lithophile element diagrams (e.g. La versus Nb; Supplementary Fig. 2a), Rairua and Anatonu inclusions exhibit a linear trend that is likely caused by the combination of partial melting and mixing from two different sources (i.e. HIMU and

depleted components)[9-11]. In plots of incompatible lithophile elements (e.g. La) against Cl, the majority of melt inclusions define such a linear trend because Cl behaves as an incompatible element (Supplementary Fig. 2b). However, a small number of inclusions show high Cl for a given La concentration, and hence plot above the compositional trend defined by the majority of inclusions. We suspect that such excessive Cl unrelated to lithophile incompatible elements is ascribed to assimilation and therefore we excluded the four melt inclusions with Cl/La > 25 from the discussion and figures in the main text (RAV-08 OL-10 MI-01, RAV-14 OL-01 MI-01, RAV27 OL-05 MI-01 and RAV-30 OL-29 MI-03 (shown by open symbols in Supplementary Fig. 2). We also excluded one Rairua inclusion (RAV-33 OL-11 MI-01) from the discussion and figures in the main text because it has anomalously high concentrations of incompatible and rare earth elements (e.g. La; 168 p.p.m.).

The Rairua melt inclusions showed a negative correlation for Cl/Nb and for Cl/K with $^{207}Pb/^{206}Pb$ (Figs. 1b and 1c). As discussed in the main text, this correlation cannot be explained by assimilation of seawater, brine, altered oceanic crust, or sediments because such contaminants have $^{207}Pb/^{206}Pb$ values similar to or higher (i.e. less radiogenic Pb isotopes) than those of the Anatonu inclusions (Fig. 1a)[12,13]. Indeed, Hauff et al. (ref. 13) reported small-scale Pb isotopic heterogeneity in aged (>130 Ma) altered oceanic crust. Vein materials containing secondary minerals such as calcite and smectite have highly radiogenic Pb isotopes because of recent increases in U/Pb and Th/Pb and subsequent radiogenic ingrowth (Supplementary Fig. 1b). However, bulk altered oceanic crust does not have such radiogenic Pb isotopes because these materials are volumetrically small (<10%)[13]. Even if the vein materials were selectively assimilated by magmas, this effect should modify Pb isotopic compositions oblique to the trend defined

by the melt inclusions from Raivavae (Rairua and Anatonu) and Mangaia (Supplementary Fig. 1b). We conclude that shallow assimilation cannot account for radiogenic Pb isotopes in Rairua and Mangaia melt inclusions and that elevated Cl/Nb and Cl/K coupled with radiogenic Pb isotopes should be characteristic of the HIMU mantle source. Based on Cl/Nb and Cl/K, type-2 inclusions reported previously[2] correspond to the Rairua inclusions. Although the previous authors[2] did not measure the Pb isotopic composition of the inclusions, the single host rock including the type-2 inclusions has radiogenic Pb isotopes. Similarly, type-1 inclusions likely correspond to the Anatonu inclusions because the host rocks of those type-1 inclusions have less radiogenic Pb isotopes.

The isotopic variation of Raivavae host basalts is best explained by binary mixing of melts from HIMU and depleted components[9-11]. Although the elemental composition and lithology of the HIMU source have been poorly understood, we simply assume 2 Ga oceanic crust as the HIMU source material for the purpose of drawing the mixing lines between the two different melts (Figs. 1 and 2) using geochemical compositions of the mixing components and partition coefficients given in Supplementary Table 3. The HIMU melt composition was calculated by assuming a large-degree (30%) of non-modal fractional melting of the eclogitic oceanic crust for which Cl concentration was given as 207 p.p.m. for the bulk oceanic crust and 60 p.p.m. for that with partial Cl loss after dehydration[14-17]. As discussed in the main text, the HIMU melt likely was produced from carbonated sources. High-field strength elements, including Hf, are much less soluble in carbonated melts than in silicate melts, whereas rare earth elements and large-ion lithophile elements remain incompatible[18]. Because partition coefficients for the carbonated eclogite system are not available thus far, we assumed Hf partitioning between carbonated melt and eclogite to be unity. The present-day $^{207}Pb/^{206}Pb$

of the HIMU source is calculated by assuming 2 Ga source age after Stracke et al. (ref. 19)

The nature of the depleted component is less well constrained, but it is slightly enriched in isotopic composition relative to normal MORBs[9]. Lassiter et al. (ref. 9) suggested that this component received a contribution from enriched veins sampled by low-degree partial melting of the ambient mantle. Konter et al. (ref. 20) have suggested the ubiquitous presence of the Common (C) mantle component[21] in the source region of the Austral–Cook Islands. Because of the uncertain composition of source material, Hanyu et al. (ref. 22) used the composition of the normal MORB source as the proxy of the depleted component. They suggested that low-degree partial melting of the depleted component is requisite to reproduce the heavy rare earth element compositions. For simplicity, we assumed that the melt from the depleted component was produced by 1% non-modal fractional melting of the MORB mantle source with 0.51 p.p.m. Cl (ref. 23) and used bulk partition coefficients between basaltic melt and garnet peridotite (Supplementary Table 3)[22-24].

The calculated mixing lines account for the higher Cl/K, Cl/Nb and Nd/Hf and for the lower K/U in Rairua melt inclusions with radiogenic Pb isotopes than in Anatonu inclusions with less radiogenic Pb isotopes, if Cl is partially lost from the subducted oceanic crust and Hf is only weakly partitioned into the carbonated melt. However, compared to Rairua melt inclusions, Mangaia inclusions have more radiogenic Pb isotopes despite having similar ranges of Cl/K, Cl/Nb, K/U and Nd/Hf. This implies a geochemically inhomogeneous HIMU mantle source impregnated with subducted oceanic crust having various recycling ages or extent of (U+Th)/Pb differentiation.

Supplementary References

1 Michael, P. J. & Cornell, W. C. Influence of spreading rate and magma supply on crystallization and assimilation beneath mid-ocean ridges: Evidence from chlorine and major element chemistry of mid-ocean ridge basalts. *J. Geophys. Res.* **103**, 18325-18356 (1998).

2 Lassiter, J. C., Hauri, E. H., Nikogosian, I. K. & Barsczus, H. G. Chlorine-potassium variations in melt inclusions from Raivavae and Rapa, Austral Islands: constraints on chlorine recycling in the mantle and evidence for brine-induced melting of oceanic crust. *Earth Planet. Sci. Lett.* **202**, 525-540 (2002).

3 Stroncik, N. A. & Haase, K. M. Chlorine in oceanic intraplate basalts: Constraints on mantle sources and recycling processes. *Geology* **32**, 945-948 (2004).

4 Wanless, V. D. *et al.* Volatile abundances and oxygen isotopes in basaltic to dacitic lavas on mid-ocean ridges: The role of assimilation at spreading centers. *Chem. Geol.* **287**, 54-65 (2011).

5 Cabral, R. A. *et al.* Volatile cycling of H_2O, CO_2, F, and Cl in the HIMU mantle: A new window provided by melt inclusions from oceanic hot spot lavas at Mangaia, Cook Islands. *Geochem., Geophys., Geosyst.* **15**, 4445-4467 (2014).

6 Kendrick, M. A. *et al.* Contrasting behaviours of CO_2, S, H_2O and halogens (F, Cl, Br, and I) in enriched-mantle melts from Pitcairn and Society seamounts. *Chem. Geol.* **370**, 69-81 (2014).

7 Kendrick, M. A., Jackson, M. G., Hauri, E. H. & Phillips, D. The halogen (F, Cl, Br, I) and H_2O systematics of Samoan lavas: Assimilated-seawater, EM2 and high-$^3He/^4He$ components. *Earth Planet. Sci. Lett.* **410**, 197-209 (2015).

8 Shimizu, K. *et al.* Two-component mantle melting-mixing model for the generation of mid-ocean ridge basalts: Implications for the volatile content of the Pacific upper mantle. *Geochim. Cosmochim. Acta* **176**, 44-80 (2016).

9 Lassiter, J. C., Blichert-Toft, J., Hauri, E. H. & Barsczus, H. G. Isotope and trace element variations in lavas from Raivavae and Rapa, Cook-Austral islands: constraints on the nature of HIMU- and EM-mantle and the origin of mid-plate volcanism in French Polynesia. *Chem. Geol.* **202**, 115-138 (2003).

10 Maury, R. C. *et al.* Temporal evolution of a Polynesian hotspot: New evidence from Raivavae (Austral islands, South Pacific ocean). *Bull. Soc. Geol. Fra.* **184**, 557-567 (2013).

11 Miyazaki, T. *et al.* Clinopyroxene and bulk rock Sr–Nd–Hf–Pb isotope compositions of Raivavae ocean island basalts: Does clinopyroxene record early

stage magma chamber processes? *Chem. Geol.* **482**, 18-31 (2018).

12 Frank, M. Radiogenic Isotopes: Tracers of past ocean circulation and erosional input. *Rev. Geophys.* **40**, 1-1-1-38 (2002).

13 Hauff, F., Hoernle, K. & Schmidt, A. Sr-Nd-Pb composition of Mesozoic Pacific oceanic crust (Site 1149 and 801, ODP Leg 185): Implications for alteration of ocean crust and the input into the Izu-Bonin-Mariana subduction system. *Geochem. Geophys. Geosyst.* **4**, 8913, doi:10.1029/2002gc000421 (2003).

14 Becker, H., Jochum, K. P. & Carlson, R. W. Trace element fractionation during dehydration of eclogites from high-pressure terranes and the implications for element fluxes in subduction zones. *Chem. Geol.* **163**, 65-99 (2000).

15 Barnes, J. D., Manning, C. E., Scambelluri, M. & Selverstone, J. in *The Role of Halogens in Terrestrial and Extraterrestrial Geochemical Processes: Surface, Crust, and Mantle* (eds. Harlov, D. E. & Aranovich, L.) 545-590 (Springer International Publishing, 2018).

16 Barnes, J. D. & Cisneros, M. Mineralogical control on the chlorine isotope composition of altered oceanic crust. *Chem. Geol.* **326-327**, 51-60 (2012).

17 Marschall, H. R., Altherr, R., Gméling, K. & Kasztovszky, Z. Lithium, boron and chlorine as tracers for metasomatism in high-pressure metamorphic rocks: a case study from Syros (Greece). *Mineral. Petrol.* **95**, 291, doi:10.1007/s00710-008-0032-3 (2009).

18 Dasgupta, R., Hirschmann, M. M., McDonough, W. F., Spiegelman, M. & Withers, A. C. Trace element partitioning between garnet lherzolite and carbonatite at 6.6 and 8.6 GPa with applications to the geochemistry of the mantle and of mantle-derived melts. *Chem. Geol.* **262**, 57-77 (2009).

19 Stracke, A., Bizimis, M. & Salters, V. J. M. Recycling oceanic crust: Quantitative constraints. *Geochem. Geophys. Geosyst.* **4**, 8003, doi:10.1029/2001GC000223 (2003).

20 Konter, J. G. *et al.* One hundred million years of mantle geochemical history suggest the retiring of mantle plumes is premature. *Earth Planet. Sci. Lett.* **275**, 285-295 (2008).

21 Hanan, B. B. & Graham, D. W. Lead and helium isotope evidence from oceanic basalts for a common deep source of mantle plumes. *Science* **272**, 991-995 (1996).

22 Hanyu, T. *et al.* Geochemical diversity in submarine HIMU basalts from Austral Islands, French Polynesia. *Contrib. Mineral. Petrol.* **166**, 1285-1304 (2013).

23 Salters, V. J. M. & Stracke, A. Composition of the depleted mantle. *Geochem. Geophys. Geosyst.* **5**, Q05004, doi:10.1029/2003GC000597 (2004).

24 Stracke, A. & Bourdon, B. The importance of melt extraction for tracing mantle heterogeneity. *Geochim. Cosmochim. Acta* **73**, 218-238 (2009).

25 Yurimoto, H. *et al.* Lead isotopic compositions in olivine-hosted melt inclusions from HIMU basalts and possible link to sulfide components. *Phys. Earth Planet. Inter.* **146**, 231-242 (2004).

26 Saal, A. E., Hart, S. R., Shimizu, N., Hauri, E. H. & Layne, G. D. Pb Isotopic variability in melt Inclusions from oceanic island basalts, Polynesia. *Science* **282**, 1481-1484 (1998).

27 Saal, A. E. *et al.* Pb isotopic variability in melt inclusions from the EMI–EMII–HIMU mantle end-members and the role of the oceanic lithosphere. *Earth Planet. Sci. Lett.* **240**, 605-650 (2005).

28 Paul, B. *et al.* Melt inclusion Pb-isotope analysis by LA–MC-ICPMS: Assessment of analytical performance and application to OIB genesis. *Chem. Geol.* **289**, 210-223 (2011).

29 Shimizu, K. *et al.* H_2O, CO_2, F, S, Cl, and P_2O_5 analyses of silicate glasses using SIMS: Report of volatile standard glasses. *Geochem. J.* **51**, 299-313 (2017).

30 Jochum, K. P. *et al.* GeoReM: A New Geochemical Database for Reference Materials and Isotopic Standards. *Geostand. Geoanal. Res.* **29**, 333-338 (2005).

31 Sharp, Z. D. & Draper, D. S. The chlorine abundance of Earth: Implications for a habitable planet. *Earth Planet. Sci. Lett.* **369–370**, 71-77 (2013).

32 Workman, R. K. & Hart, S. R. Major and trace element composition of the depleted MORB mantle (DMM). *Earth Planet. Sci. Lett.* **231,** 53-72 (2005).

33 Saal, A. E., Hauri, E. H., Langmuir, C. H. & Perfit, M. R. Vapour undersaturation in primitive mid-ocean-ridge basalt and the volatile content of Earth's upper mantle. *Nature* **419**, 451-455 (2002).

34 Urann, B. M. *et al.* Fluorine and chlorine in mantle minerals and the halogen budget of the Earth's mantle. *Contrib. Mineral. Petrol.* **172**, 51, doi:10.1007/s00410-017-1368-7 (2017).

35 Kendrick, M. A. *et al.* Seawater cycled throughout Earth/'s mantle in partially serpentinized lithosphere. *Nat. Geosci.* **10**, 222-228 (2017).

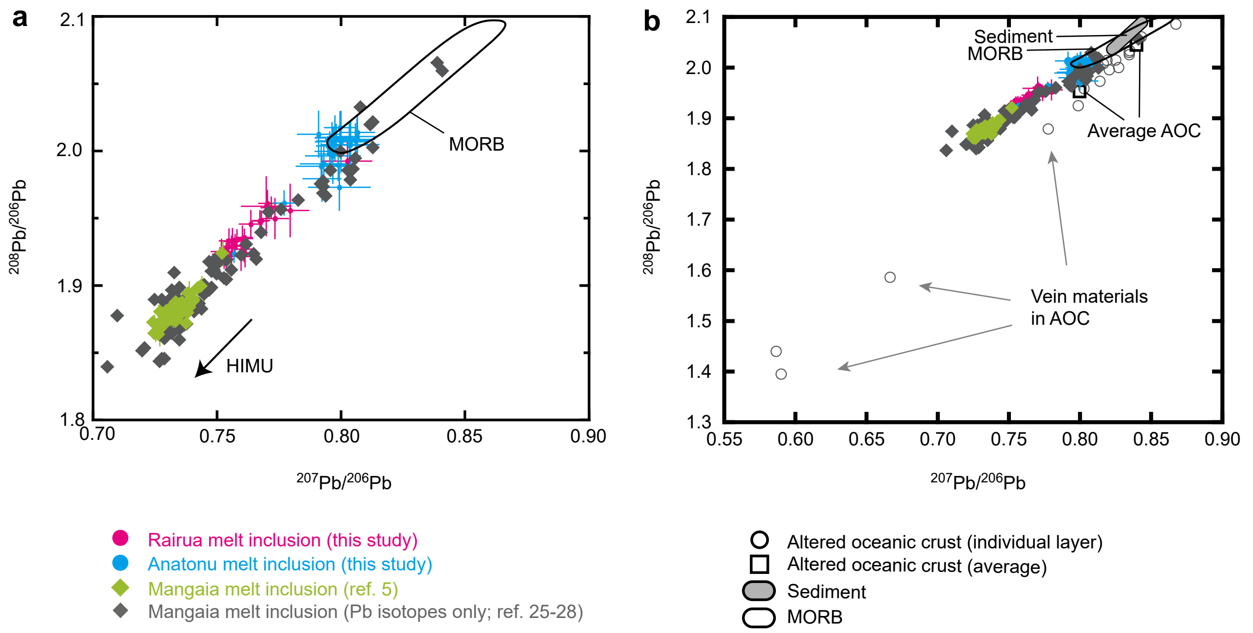

Supplementary Figure 1: Pb isotopic compositions of melt inclusions and possible contaminants. a, Melt inclusions from Raivavae (this study) and Mangaia[5]. Pb isotopes without volatile compositions are from ref. 25-28. **b**, Altered oceanic crust (AOC) and sediments from the ODP Sites 801 and 1149 as possible assimilation sources[13]. Open circles and squares indicate AOC compositions as individual layers and averages. Since ~130 Ma, radiogenic ingrowth has caused highly radiogenic Pb isotopic compositions (i.e. low $^{207}Pb/^{206}Pb$ and $^{208}Pb/^{206}Pb$) in vein materials that include secondary minerals in AOC, forming a trend that is at an angle to that defined by the Raivavae and Mangaia melt inclusions. The isotopic range of MORB is shown for comparison. Error bars with the $^{207}Pb/^{206}Pb$ and $^{208}Pb/^{206}Pb$ data of this study are bracketing standards propagated in-run 2 S.E.

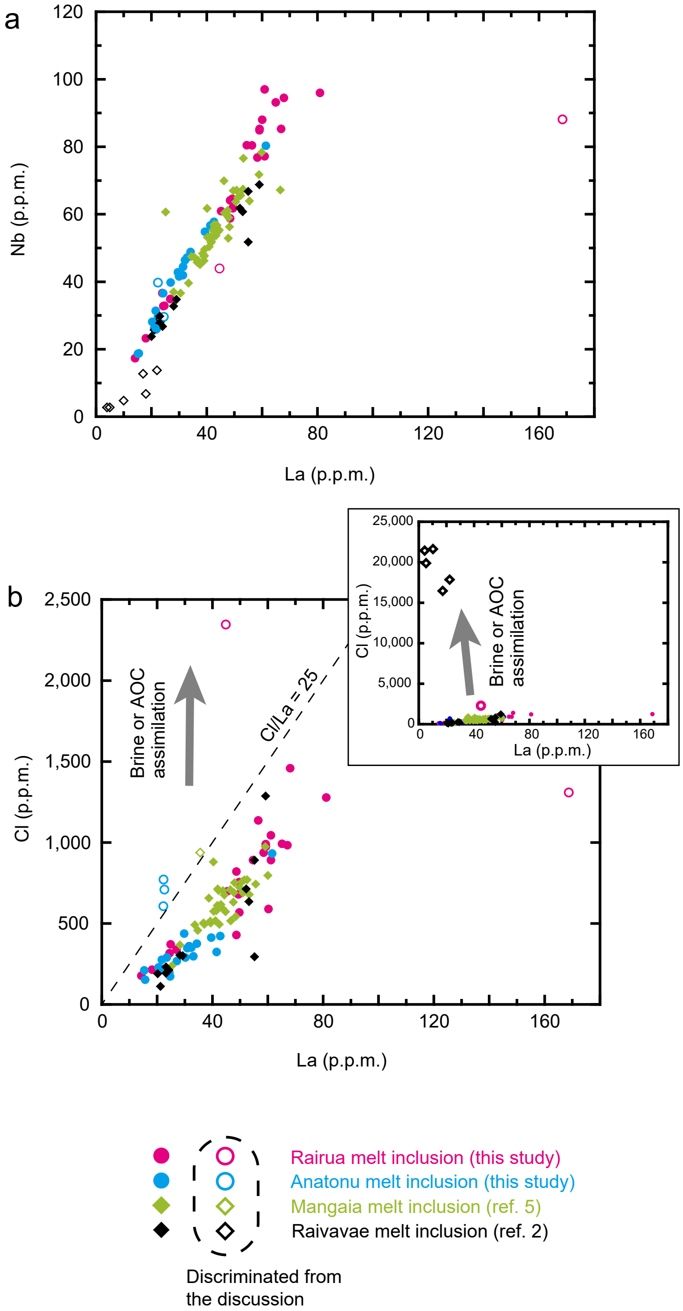

Supplementary Figure 2: Diagrams showing possible secondary Cl contamination. a, Lanthanum concentration versus Nb concentration. **b**, Lanthanum concentration versus Cl concentration. Incompatible element concentrations, including La, Nb and Cl, are positively correlated with each other. However, some melt inclusions have an excessively high Cl concentration for a given La concentration. Melt inclusions for which Cl/La > 25 (shown by open symbols) are excluded from the discussion because of possible Cl contamination from brine or brine-impregnated altered oceanic crust.

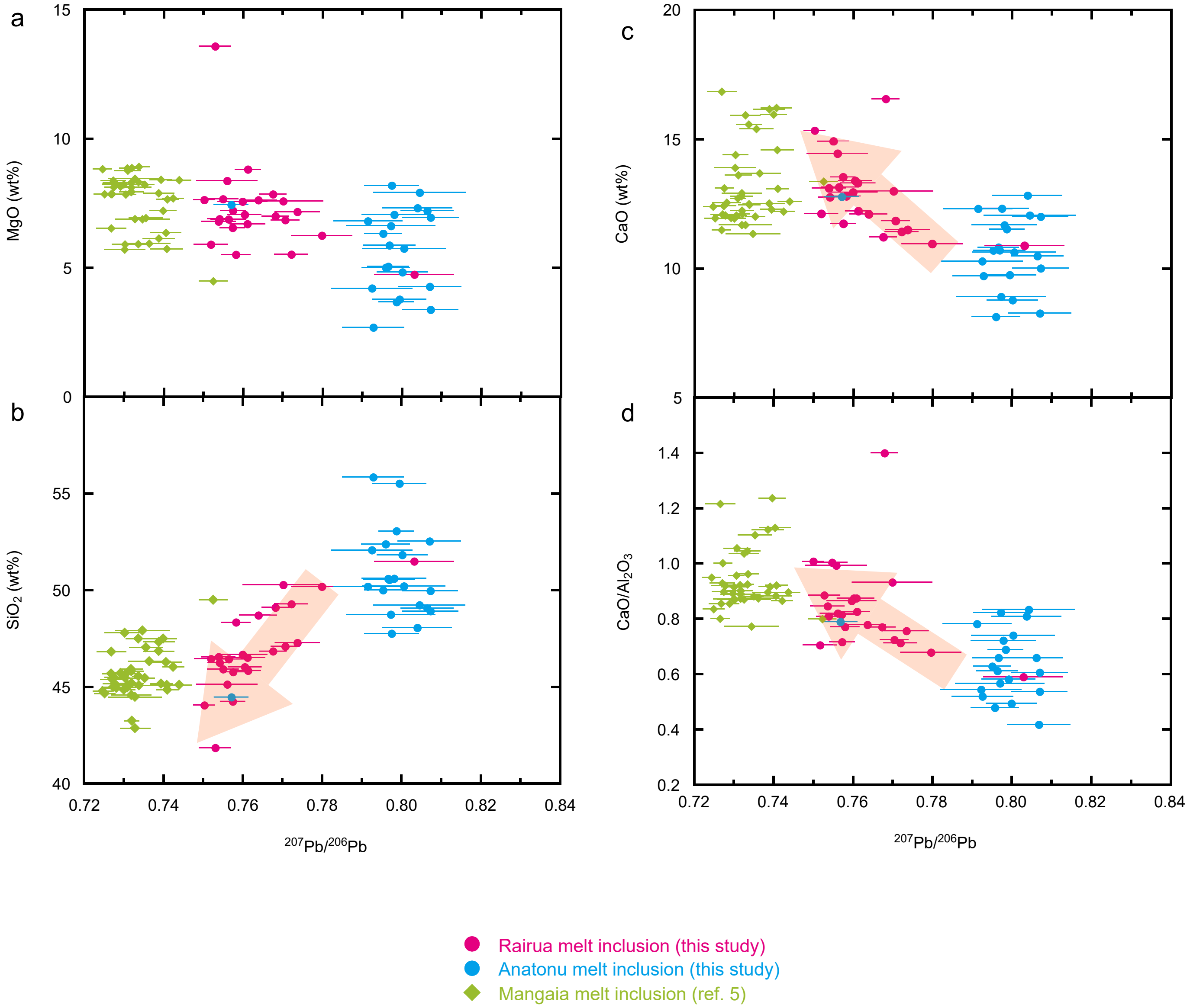

Supplementary Figure 3: $^{207}Pb/^{206}Pb$ versus major element compositions. a, $^{207}Pb/^{206}Pb$ versus MgO content. **b,** $^{207}Pb/^{206}Pb$ versus SiO_2 content. **c,** $^{207}Pb/^{206}Pb$ versus CaO content. **d,** $^{207}Pb/^{206}Pb$ versus CaO/Al_2O_3 ratio. Rairua and Anatonu melt inclusions from Raivavae (this study) and from Mangaia[5] are shown. While MgO in Rairua inclusions does not systematically change with $^{207}Pb/^{206}Pb$, these inclusions have lower SiO_2, higher CaO and higher CaO/Al_2O_3 at low values of $^{207}Pb/^{206}Pb$. Error bars with the $^{207}Pb/^{206}Pb$ data of this study are bracketing standards propagated in-run 2 S.E.

a

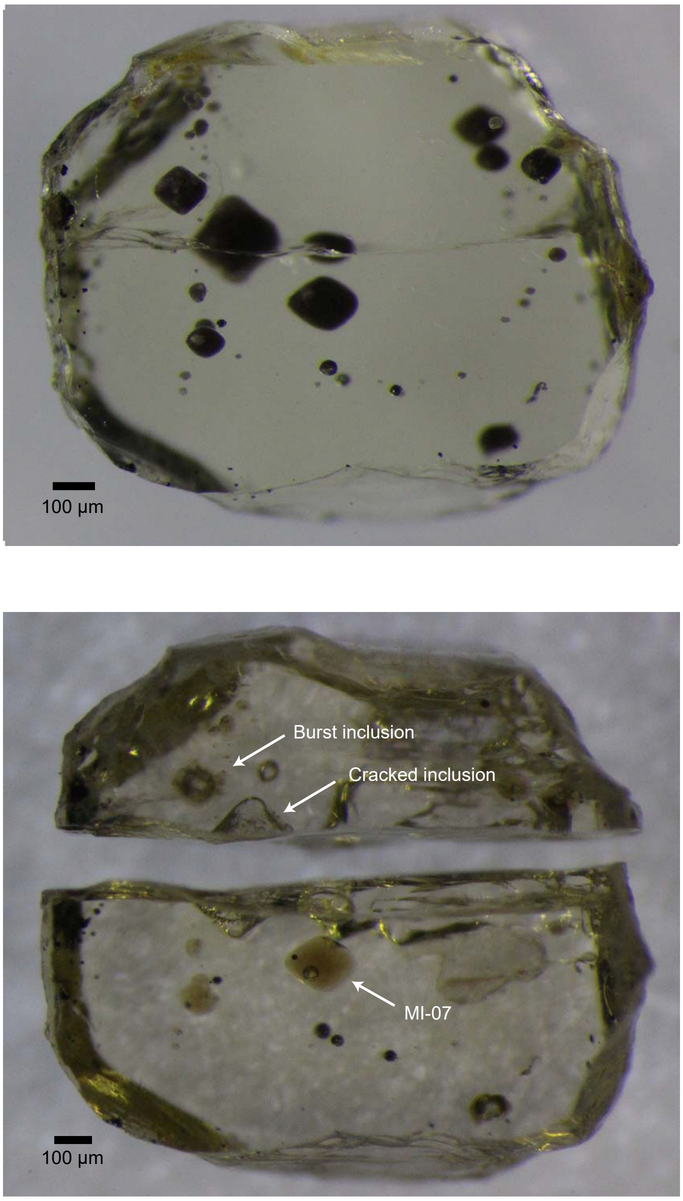

b

Supplementary Figure 4: Photomicrographs of unheated and heated olivine containing melt inclusions. a, Polished olivine (RAV-30_OL-07) before heating. **b**, Same olivine after heating. Inclusions that burst or cracked during heating experiments were disregarded. Rehomogenised melt inclusion (MI-07) was exposed by further polishing for *in-situ* geochemical analysis.

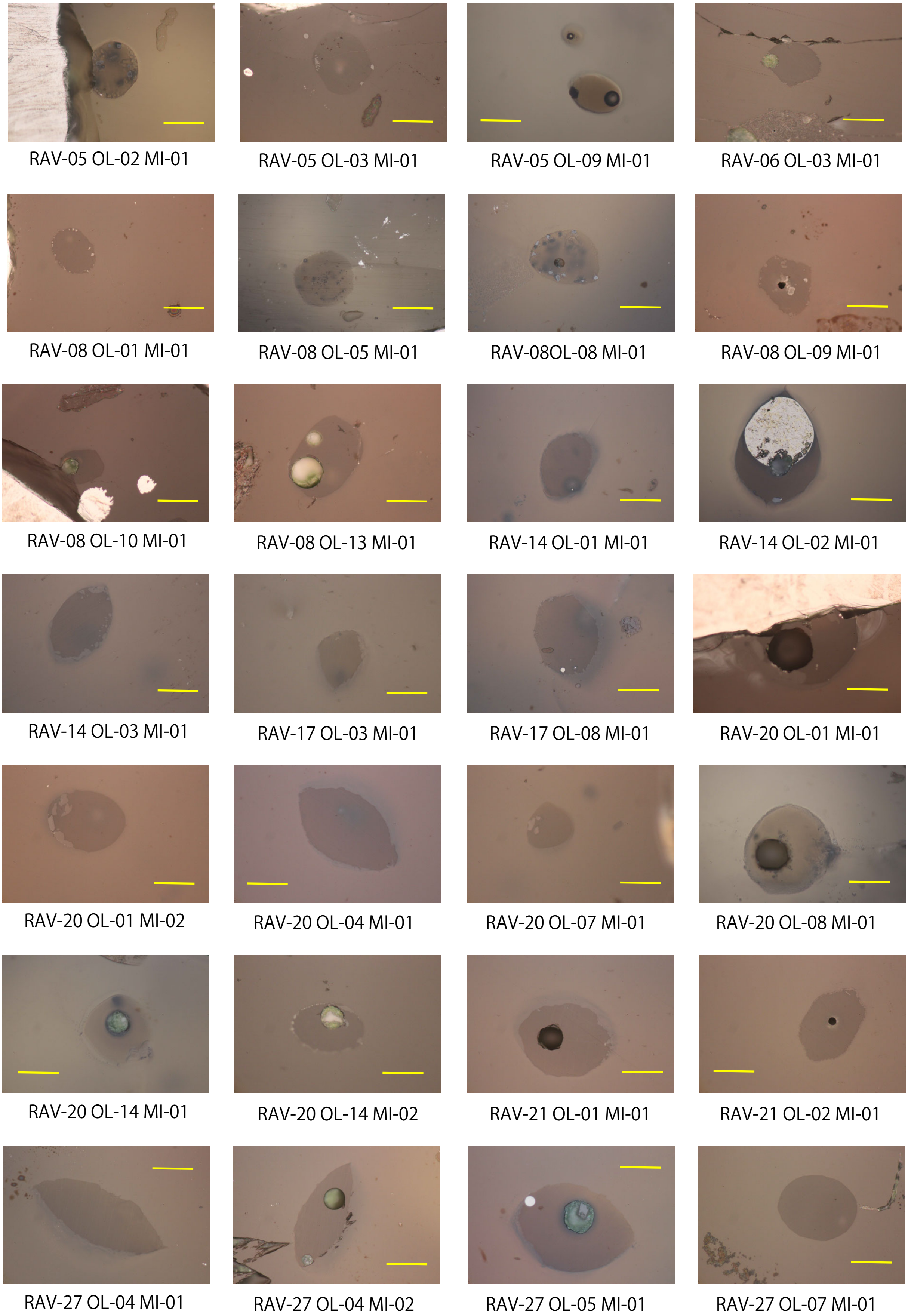

Supplementary Figure 5 (continued on next page)

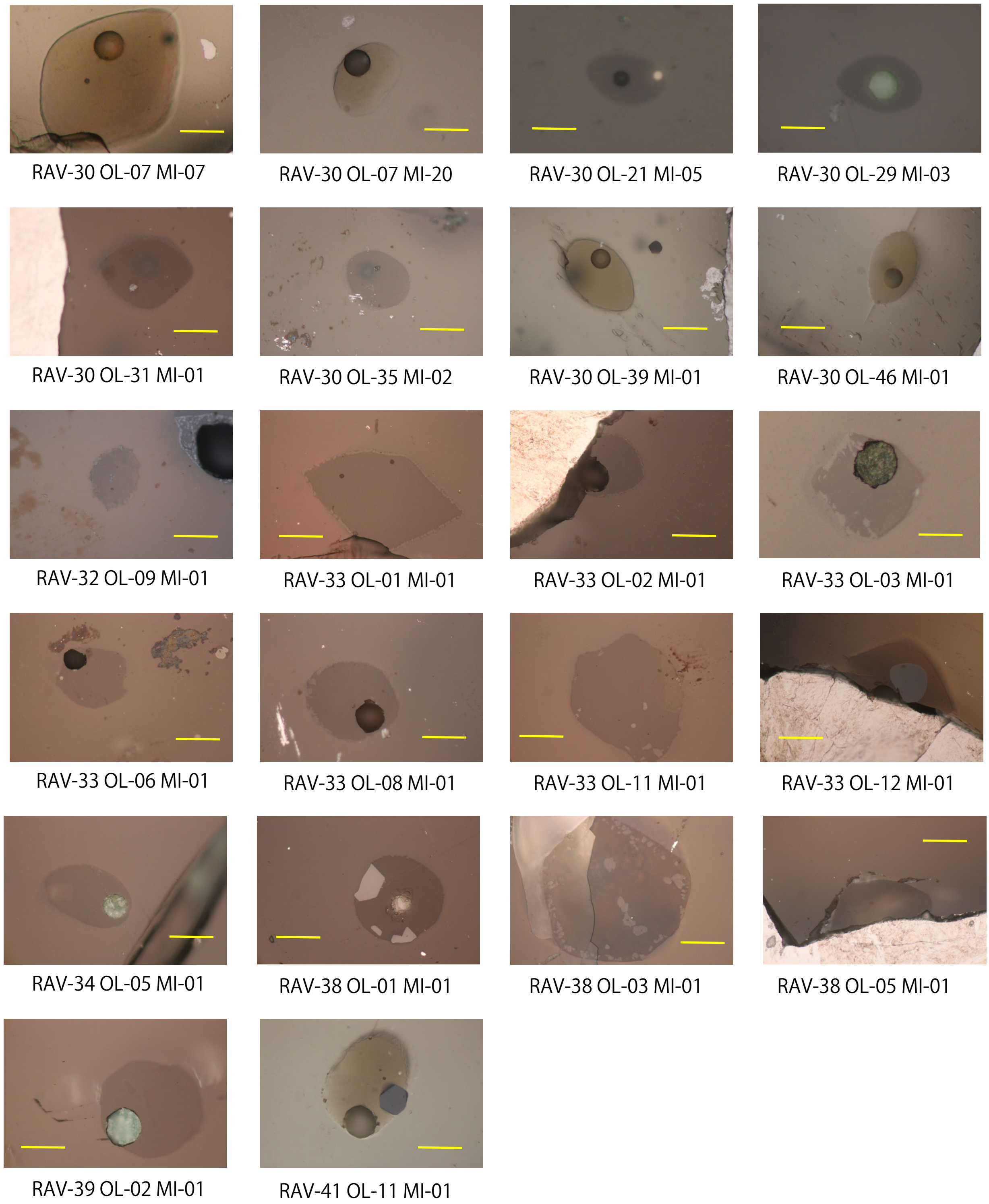

Supplementary Figure 5: Photomicrographs of rehomogenised melt inclusions. Photomicrographs were taken after host olivines were polished and mounted on indium discs but prior to final polishing and cleaning for SIMS analysis. Some melt inclusions were cracked during polishing or pressing onto the discs. We avoided any cracked part, former shrinkage bubbles, spinels and edges of melt inclusions for all measurements except for the samples RAV-08 OL-10 MI-01 and RAV-20 OL-07 MI-01, for which laser spots for Pb isotope analysis partially overlap the surrounding host olivines because otherwise there was not enough room for laser ablation (~30 μm diameter). The scale bar equals 50 μm.

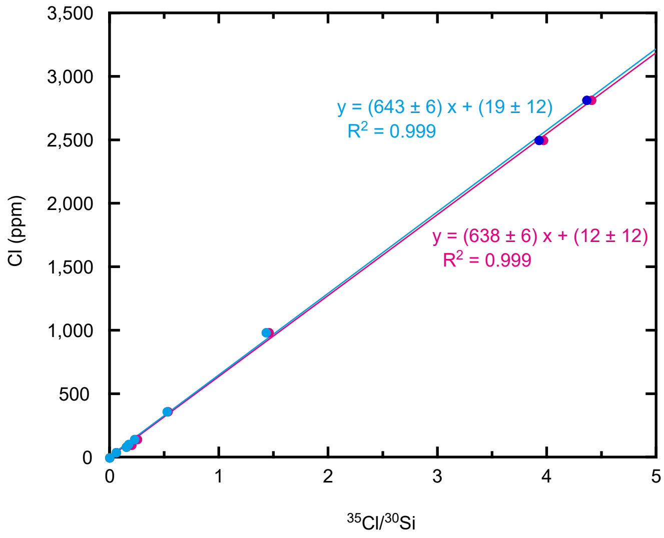

Supplementary Figure 6: Chlorine calibration lines by SIMS analyses. Secondary ion intensity of ^{35}Cl was normalised by that of ^{30}Si. Regression lines were calculated by applying the least squares method for the data of international and in-house standards with basaltic compositions[29]. Calibration lines were determined before each analytical session (purple line, 9 November 2015; light-blue line, 14 June 2016). Regression formulae with 1 S.D. (standard deviation) and coefficient of determination are shown for each calibration line.

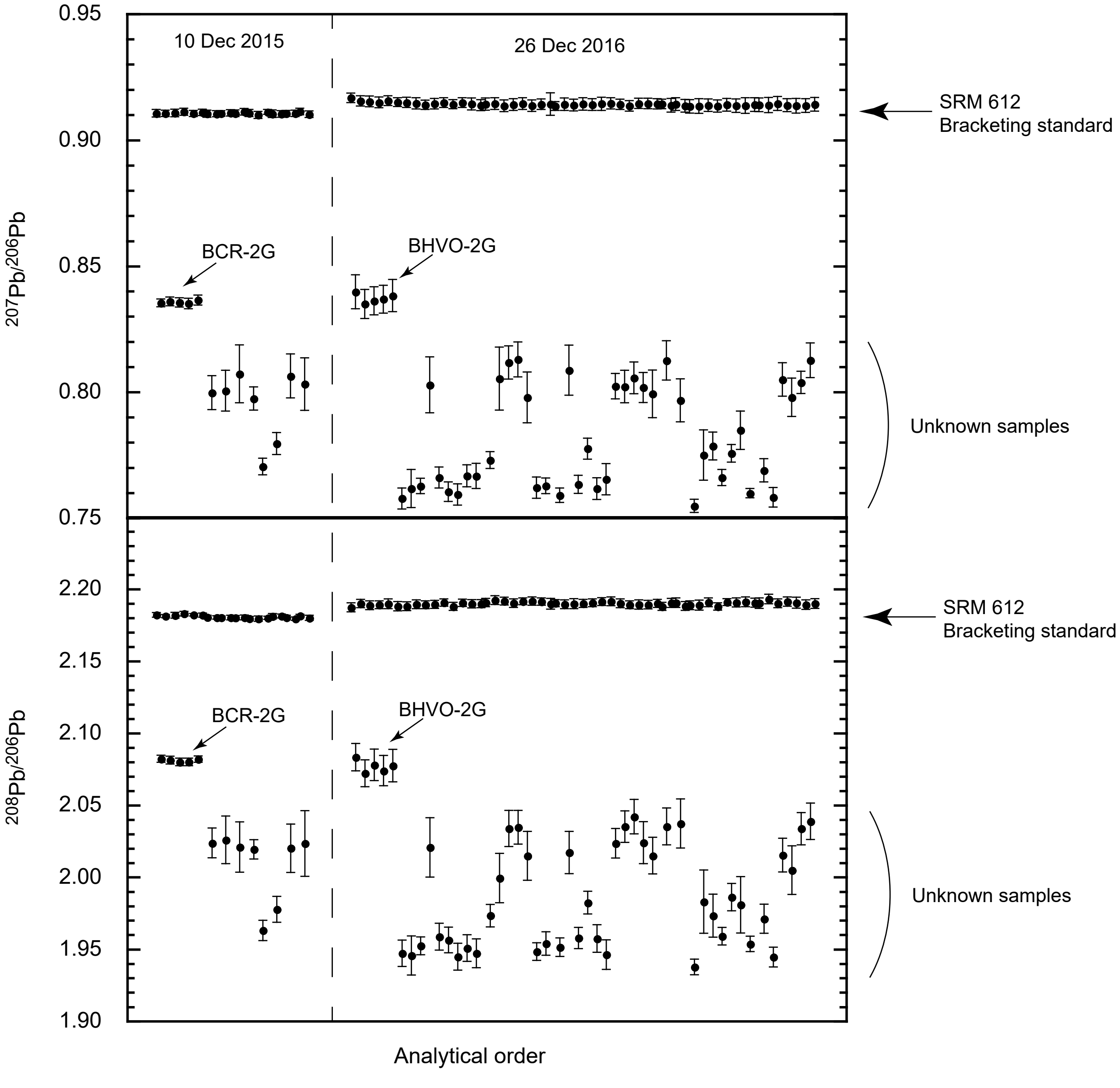

Supplementary Figure 7: $^{207}Pb/^{206}Pb$ and $^{208}Pb/^{206}Pb$ of bracketing standards, international standards and unknown samples shown in analytical order. Measurements of Pb isotopes were performed on two separate days. Error bars with the $^{207}Pb/^{206}Pb$ and $^{208}Pb/^{206}Pb$ data are in-run 2 S.E. Isotope ratios of international standards (BCR-2G and BHVO-2G) and unknown samples shown in the figure are the measured values before external mass bias correction using bracketing standards.
