# Supplementary material for: Tiny droplets of ocean island basalts unveil Earth’s deep chlorine cycle
Source: Nat Commun. 2019 Jan 4;10:60. doi: 10.1038/s41467-018-07955-8 (PMC6320363; doi:10.1038/s41467-018-07955-8)
Supplement: Supplementary file 3 — Description of Additional Supplementary Files [file 41467_2018_7955_MOESM3_ESM.pdf]

Description of Additional Supplementary Files

File Name: Supplementary Table 1
Description: Chemical compositions and Pb isotope ratios of melt inclusions from Raivavae.

File Name: Supplementary Table 2
Description: Measured compositions of standard samples.

File Name: Supplementary Table 3
Description: Chemical compositions and partition coefficients used for melt mixing model.

File Name: Supplementary Table 4
Description: Model chlorine inventory in the Earth.
